# Nutritional Strategies to Promote Bovine Oocyte Quality for In Vitro Embryo Production: Do They Really Work?

**DOI:** 10.3390/vetsci10100604

**Published:** 2023-10-04

**Authors:** Miguel A. Velazquez

**Affiliations:** School of Natural and Environmental Sciences, Newcastle University, Newcastle Upon Tyne NE1 7RU, UK; miguel.velazquez@ncl.ac.uk

**Keywords:** oocyte competence, nutrition, preimplantation embryo, in vitro culture, cattle

## Abstract

**Simple Summary:**

The nutritional status of oocyte donors plays a significant role in the efficiency of in vitro embryo production (IVEP) in cattle. The available evidence suggests that oocyte donors with a moderate body condition score (i.e., 3 in a 1–5 scale) subjected to ovum pick have an increased chance of providing a metabolic microenvironment in ovarian follicles that will promote embryo formation in vitro. The usefulness of fatty acid and micronutrient supplementation to improve the IVEP of oocyte donors is debatable with the current information available. As such, the supply of maintenance nutritional requirements according to developmental and productive stage seems to be enough to provide bovine oocyte donors with a good chance of producing embryos in vitro.

**Abstract:**

The ability of bovine oocytes to reach the blastocyst stage (i.e., embryo with around 150 cells in cattle) in vitro can be affected by technical (e.g., culture medium used) and physiological factors in oocyte donors (e.g., age, breed). As such, the nutritional status of oocyte donors plays a significant role in the efficiency of in vitro embryo production (IVEP), and several nutritional strategies have been investigated in cattle subjected to ovum pick-up (OPU). However, there is no clear consensus on the reliability of nutritional schemes to improve IVEP in cattle. Available evidence suggests that a moderate body condition score (i.e., 3 in a 1–5 scale) in cattle is compatible with a metabolic microenvironment in ovarian follicles that will promote embryo formation in vitro. The usefulness of fatty acid and micronutrient supplementation to improve IVEP in cattle is debatable with the current information available. Overall, the supply of maintenance nutritional requirements according to developmental and productive stage seems to be enough to provide bovine oocyte donors with a good chance of producing embryos in vitro. Future nutrition research in cattle using OPU-IVEP models needs to consider animal well-being aspects (i.e., stress caused by handling and sampling), which could affect the results.

## 1. Introduction

**Figure 1:**
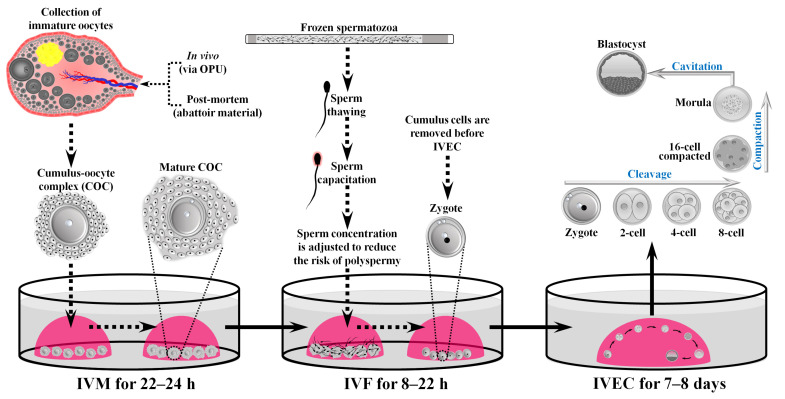
Main steps in standard in vitro production of embryos for commercial purposes in cattle. Oocytes are usually collected via ovum pick-up (OPU) on live animals. In vitro maturation (IVM) can be done in portable incubators sent overnight to the laboratory. In vitro fertilisation (IVF) and in vitro embryo culture (IVEC) are performed at the laboratory. Components are not drawn to scale.

The growing global human population, predicted to surpass 9 billion by 2050 [1], coupled with the projected decrease in food availability (e.g., red meat) due to climate change [2], emphasises the critical role that efficient and sustainable animal production will play to achieve food security during this global challenge [3,4]. Among several animal products, the demand for cattle-derived products (e.g., milk) will continue to increase worldwide [5,6]. However, future cattle production will increasingly rely on the use of assisted reproductive technologies (ARTs), such as in vitro embryo production (IVEP) coupled with embryo transfer to reduce the number of animals involved in the production of replacement animals of high genetic merit in dairy and beef herds [5,7,8,9,10]. Indeed, the use of IVEP in cattle has been increasing during the last two decades [11], superseding other ARTs such as multiple ovulation and embryo transfer (MOET) [12]. The combination of IVEP and embryo transfer will vastly reduce the number of cows involved in specialised cattle breeding and will have a significant impact on the reduction in the carbon footprint from the cattle industry. Furthermore, IVEP will play a critical role in the implementation of genomic selection (i.e., in vitro breeding) [13] and gene editing technologies to improve efficiency in cattle production systems needed to achieve sustainable animal production [11]. Standard bovine IVEP is comprised of three main steps, in vitro maturation (IVM), in vitro fertilisation (IVF), and in vitro embryo culture (IVEC) (Figure 1).

For breeding purposes, oocytes can be collected from abattoir-derived ovaries [12], but transvaginal ultrasound-guided oocyte retrieval, a technique commonly known as “ovum pick-up” (OPU) [14,15,16], is usually applied for the in vivo collection of oocytes. Although the use of bovine IVEP is increasing worldwide, there is substantial variation among laboratories/companies in IVEP efficiency. The percentage of embryos produced from cultured oocytes collected via OPU in several studies in the last 10 years varied from 14 to 60% (i.e., maximum production reported per study), with few cases reporting blastocyst production over 50% (Table 1). This is comparable to some extend with the 15–31% in vitro embryo production from data compiled from the American Embryo Transfer Association (AETA) [14] and the 20–32% in vitro blastocyst production reported from data generated at Brazilian farms [17]. The variation in bovine IVEP efficiency is multifactorial, including factors such as lactational status [18], oocyte donor age [16,19,20] and breed [21,22,23,24], bull (including bull breed) [17,25,26], heat stress [27], interval between OPU sessions [28], use of ovarian stimulation [14,29], inherent antral follicle number [30,31], culture media [32,33], equipment [34,35,36], and nutrition of the oocyte donor [37,38]. Of these factors, the nutritional status of oocyte donors has been investigated substantially given the critical role that nutrition plays in fertility. However, a clear consensus on the reliability of nutritional schemes to improve IVEP outcome in cattle based on well-established evidence is not available. In this review, the nutritional strategies of bovine oocyte donors to promote oocyte developmental competence (i.e., ability of the oocyte to reach the blastocyst stage) in vitro will be discussed, with the aim of providing an overview of the usefulness of such strategies.

## 2. Impact of Dietary Intake of Bovine Oocyte Donors on In Vitro Embryo Production

Meeting the nutritional requirements for specific developmental and productive phases is essential for reproductive success in cattle [64,65]. A common approach to evaluate nutritional status is the measurement of body condition score (BCS) in both dairy and beef cattle [64,66,67]. An OPU-IVEP model found that oocytes from beef × dairy heifers with a low BCS (2 in a 5-point scale, 1 = lean, 5 = obese) produced fewer blastocysts when compared with oocytes from heifers with a BCS of 3–3.5 [37]. In contrast, a more recent study comparing Holstein–Friesian cows with a BCS of 1 to 3 (also using a 5-point scale) did not observe differences in in vitro blastocyst production with abattoir-derived oocytes [68]. Furthermore, it was also reported that cows with a BCS of 1 produced embryos with a higher number of blastomeres compared with cows with a BCS of 2–3 [68]. An increased number of cells in blastocysts has also been reported in models of undernutrition in superovulated sheep [69] and cattle [70]. This response to undernutrition could represent a mechanism aimed at protecting the oocyte in the developing follicle (and embryo in the reproductive tract) from nutritional deficits [71]. Still, given the well-documented detrimental effects of low BCS on bovine embryo production [71,72,73], it is difficult to reconcile the lack of effect of very-low BCS on oocyte developmental competence [68]. These contrasting results could be partially related to the low blastocyst production achieved from oocytes cleaved (14.7 to 18.9%) in experimental groups in the latter study, suggesting that in vitro conditions can sometimes mask the effects of BCS on oocyte developmental competence [68]. Nevertheless, a previous study using a similar experimental approach reported impaired IVEP in Holstein × Zebu cows with a BCS of 1 compared with oocyte donors with a BCS of 2–3 [74]. Moreover, dairy cows with a BCS of 1.5–2.5 produced oocytes with a lower capacity to achieve the blastocyst stage in vitro than oocytes from cows with a BCS of 3–4 [75].

Level of dietary energy can affect oocyte developmental competence, as shown in Holstein heifers fed with 1.8 of their maintenance energy requirements (M) for 32 days, resulting in decreased blastocyst production in vitro when compared to heifers fed with a 0.8 M diet [76]. This negative effect of a high energy intake on blastocyst formation was associated with high urea concentrations in blood, which are known to be detrimental for oocyte developmental competence [77]. In beef × dairy heifers with a low BCS of 2, an improvement in their in vitro embryo production was observed when they were fed with a 2 M diet that increased their BCS to 3. However, in heifers with a moderate BCS of 3.5, in vitro embryo production was decreased when they reached a BCS of 4 after being fed with a 2 M diet [37]. In contrast, the overfeeding of Holstein heifers for 16 weeks did not affect negatively their IVEP compared to overfed heifers that were switched to feed restriction for the second half of the experimental period (i.e., 8 weeks) [38]. In the latter study, growing heifers were used (14 months of age), which reached a BCS of 3 by the end of the overfeeding period (18 months of age), suggesting that the nutritional status achieved during overfeeding did not induce a harmful metabolic microenvironment in ovarian follicles even though blood concentrations of insulin, insulin-like growth factor-1 (IGF-1), and β-hydroxybutyrate (BHB) were increased in comparison with restricted heifers [38]. There is an undetermined concentration threshold in which these metabolic hormones and metabolite can be detrimental for oocyte developmental competence [71,76]. It was suggested that further extension of the overfeeding period would have resulted in a detrimental effect on oocyte developmental competence [38], as observed in more matured heifers (20 months of age) with a moderate BCS of 3.5 that were overfed (i.e., 2 M) for three consecutive oestrous cycles, where IVEP was decreased and was associated with a high-diet induced BCS of 4 and hyperinsulinemia [37]. Indeed, in vivo embryo production in superovulated cattle is commonly impaired once BCS reaches 4 points or more (in a 1–5 scale) [78,79]. 

More recently, a 28-day overfeeding (2 M) regime in Japanese Black cows with a BCS of 3 decreased the capacity of their oocytes to cleave in vitro, and this was associated with an increase in insulin and cholesterol concentrations in blood [63]. As for insulin, there is an undetermined concentration threshold in which cholesterol becomes detrimental for oocyte developmental competence [71,80]. However, a clear decrease in in vitro blastocyst formation was not observed in the latter study, and the BCS of oocyte donors remained unchanged during the nutritional challenge [63]. Similarly, IVEP was unaltered in Holstein heifers with a BCS of 2 who were fed with either a 2 M fibre- or starch-based diet supplied twice or four times a day for around 45 days. Weekly blood concentrations of insulin and BCS were not altered by diet, but IGF-1 and leptin were increased when starch diets were provided four times a day [81]. In addition, post-prandial increases in insulin and glucagon associated with starch consumption were observed, suggesting that the metabolic alterations associated with meal frequency do not seem to impact oocyte developmental competence [81]. The same animal model was used to test the effects of different levels of starch and leucine, showing that a high intake of starch can impair in vitro blastocyst production associated with high insulin concentrations in blood. Furthermore, the negative impact of high starch consumption was alleviated when oocyte donors were fed with high levels of leucine that increased glucagon concentrations [82]. 

Interestingly, Sales et al. [22] reported that oocyte donors of *Bos indicus* origin (i.e., Gir cattle) with an average BCS of 3 subjected to a 120-day overfeeding (i.e., 1.7 M) period displayed an increase in blastocyst production during the first 60 days of high-energy diet consumption. However, blastocyst production was reduced to levels observed in control cows (i.e., 1 M) during the last 60 days of the overfeeding period, and this nutritional response to a high energy intake was not observed in Holstein cows (i.e., *Bos taurus*) [22]. The response to high energy supplementation in Gir cows was interpreted as a negative effect on IVEP caused by overfeeding [22], but it seems more akin with the response to the so-called “nutritional flushing” reported in ewes, where increased nutritional intake enhances folliculogenesis and ovulation rate [83]. Gene expression was analysed in oocytes, and the reduction in the IVEP of overfeed cows to levels observed in well-fed cows was associated with the downregulation of genes involved in the control of cellular stress (*HSP701*), energy metabolism (*SLC2A1*), and cell proliferation and survival (*IGF1R*, and *IGF2R*) [22]. It is unknown if the continuation of overfeeding for a longer period in the model used by Sales et al. [22] would cause a further reduction in IVEP, indicating a truly negative effect of overnutrition on oocyte developmental competence. The lack of the initial positive effect of a high energy intake on IVEP production in Holstein cows could be related to an increase in the antral follicle population usually present in *Bos indicus* cattle compared to *Bos taurus* breeds such as Holstein cattle [84,85]. Indeed, lower oocyte collection and IVEP has been reported in Holstein cattle compared to *Bos indicus* breeds, including Gir cattle [22,86,87]. Furthermore, oocytes from Holstein cows displayed a higher level of apoptosis compared to Gir cows [22], which could have masked the effects of energy consumption on IVEP. 

Although undernutrition does not always affect the ability of oocytes to form a blastocyst [71], there is evidence indicating that a low plane of nutrition can decrease in vitro [88,89] and in vivo [90,91,92] oocyte developmental competence in cattle and sheep. As such, some strategies have been developed to try to promote the embryo production of undernourished or metabolically compromised oocyte donors in bovine OPU-IVEP models. For example, superstimulated Holstein heifers subjected to feed restriction that were drenched once a day for nine days with propylene glycol showed an increase in their in vitro blastocyst production [44]. However, improved IVEP was only observed in heifers with high concentrations of anti-Müllerian hormone (AMH), suggesting the presence of a high antral follicle population, which resulted in a higher availability of good-quality oocytes. The treatment was associated with increased blood concentrations of insulin, IGF-1, and glucose, along with decreased levels of BHB and urea [44]. Further research with this OPU-IVEP model indicated that propylene glycol restored IGF-1 levels in follicular fluid induced by feed restriction, and normalised cumulus cell gene expression of IGF-1, IGF-1 receptor, and IGF binding protein 4 to levels found in well-fed controls. Furthermore, in vitro-produced blastocysts from propylene glycol-treated heifers displayed an increased expression of genes downregulated by feed restriction, including genes involved in energy metabolism (*SCL2A1*) and membrane permeability (*AQP3*) [93]. Similarly, the infusion of propylene glycol twice a day for 5 days increased the in vitro blastocyst production of Holstein cows during early lactation [94] and during their lactation peak [95]. However, propylene glycol treatment did not alter the IVEP of repeat breeder cows [94,95], and a clear improvement was not observed in dry cows and prepubertal heifers [94]. The latter findings suggest that bovine oocyte donors without nutrient deficits might not benefit from a glucogenic precursor treatment. Nevertheless, a previous study reported that Holstein–Friesian cows treated daily with propylene glycol during days 7–40 postpartum did not show a significant improvement in IVEP, even though the animals were treated during a period of metabolic stress induced by lactation [96]. Albeit speculative, the lack of effect of propylene glycol treatment on the latter study could have been associated with the stress imposed by the intensive blood sampling schedule carried out on the experimental animals, consisting of four sessions of serial jugular venepuncture at four different time points during approximately 35 days of experimentation. Accordingly, blood sampling can increase cortisol concentrations [97], and although cortisol can exert a positive effect on oocyte developmental competence at low levels [98], there is evidence linking increased levels of cortisol with impair oocyte quality [99,100,101,102]. Moreover, psychological stress can impair oocyte quality [103,104], and cattle handling has been proposed to affect fertility [105]. Hence, the possibility exists that a stress response to intensive sampling could have masked a positive effect of propylene glycol on oocyte developmental competence. Also, it has been suggested that inconsistent results following propylene glycol treatment could be due to the type of dairy cow used in experimental studies as metabolism of glucose during nutritional challenges can differ between Holstein strains [106].

Overall, the information available suggests that a high dietary intake of oocyte donors is more likely to become detrimental for IVEP when it is able to induce a significant gain in BCS in animals with a moderate BCS that, in turn, could increase metabolic hormones and metabolites to harmful levels. Also, it is likely that short-term nutritional treatments will affect oocyte developmental competence in donors with nutritional deficits, but this most probably will depend on the ability of nutritional interventions to alter the intrafollicular microenvironment.

## 3. Impact of Fatty Acid Supplementation of Bovine Oocyte Donors on In Vitro Embryo Production

Fatty acids are essential for cellular function and play a significant role in reproductive processes [107,108,109]. As such, fatty acid supplementation has been tested as a strategy to improve reproductive performance in dairy cows [65,110,111]. Dairy cows experience a period of negative energy balance caused by the inability to cover the high demand of nutrients imposed by high milk production, especially during early lactation. The negative energy balance creates an unfavourable intrafollicular microenvironment for oocyte developmental competence that can impair reproductive performance in dairy cattle [112,113,114]. Several studies have investigated the usefulness of fatty acid supplementation as a means to improve the oocyte developmental competence of lactating dairy cows in OPU-IVEP models. For example, supplementation with flaxseed oil increased the zygote cleavage rate in dairy cows associated with a decrease in ovarian follicular concentrations of linoleic, γ-linolenic, dihomo-γ-linolenic, arachidonic, and oleic acid [115]. However, blastocyst production (i.e., embryos available for embryo transfer) was not reported, which is a critical outcome indicating the level of success in OPU-IVEP models. This is relevant because improvements in embryo cleavage do not always result in increased blastocyst formation. Indeed, the majority of OPU-IVEP studies of fatty acid supplementation did not find an increase in the blastocyst production of dairy cows (Table 2).

A clear effect (statistically speaking) of fat supplementation on IVEP of dairy cows has been reported only in a few studies. Fouladi-Nashta et al. [117] found that rumen-inert fat increased blastocyst formation, and the resultant embryos showed an increased number of cells in the trophectoderm related to decreased non-esterified fatty acid (NEFA) concentrations in blood. A preliminary study reported that supplementation with 100 g/day of conjugated linoleic acid (CLA) can increase in vitro blastocyst yield in dairy cows when compared to controls (i.e., non-supplemented cows) and cows fed with 50 g/day CLA with a low blastocyst production (i.e., 8.7–8.8%) [124]. It has been suggested that lack of comparison with non-supplemented controls could account to a certain extent for the absence of beneficial effects following fatty acid supplementation in OPU-IVEP models [118,123], and it might partially explain the positive effect seen with CLA supplementation in dairy cows [124]. However, more recent work in beef crossbreed cows of *Bos taurus* origin do not support the use of CLA in oocyte donors as a means to increasing IVEP [125]. Hence, the use of CLA supplementation might be beneficial only for cattle experiencing a negative energy balance, as previously suggested [77]. Other studies did not find an effect on blastocyst production but reported effects on blastocyst quality. For instance, an increase in the morphological quality of blastocyst was observed in dairy cows fed fish oil [121]. However, morphological evaluation relies on a visual observation of the embryo, making the technique highly subjective and affected by the bias/experience level of the evaluator [126,127]. Indeed, the beneficial effect of the fish oil supplementation of oocyte donors on embryo morphological quality was not replicated in a later study by the same group [128]. An increased tolerance to cryopreservation has been claimed in Nellore cows supplemented with canola grains as the authors detected better hatching rates following embryo vitrification. However, the differences between treatments did not reach statistical significance [129].

Studies in heifers have been unable to detect a beneficial effect of fatty acid supplementation on IVEP outcome. For instance, supplementation with linseed or soya beans in Holstein heifers [130] and the addition of soy oil or protected fat to the diets of Nellore heifers [131] did not affect the capacity of their oocytes to reach the blastocyst stage in vitro. Likewise, no significant effects on in vitro embryo production were observed in Holstein–Friesian heifers fed with either rumen protected CLA or stearic acid (SA). Moreover, oocyte retrieval was compromised in heifers that received double the dose of CLA and SA due to the low availability of antral follicles for OPU, resulting in the lack of sufficient embryos for statistical comparisons [132]. Furthermore, a detrimental effect of fatty supplementation has been reported, where beef × dairy heifers displayed a lower in vitro blastocyst yield after being supplemented with calcium soaps of palm oil. This negative effect was detected in oocyte donors with a low BCS (i.e., 2.5), but not in animals with a moderate BCS (i.e., 3.0) [133]. Interestingly, the quality of resultant embryos was affected differently depending on whether the fat-supplemented oocyte donor was fed with fibre or starch. As such, blastocysts from starch-fed donors showed a low cell number, whereas embryos from fibre-fed animals presented an increased number of blastomeres [133]. It is unknown whether these divergent embryonic phenotypes represent a detrimental or beneficial effect of fat supplementation, especially when considering that the level of apoptosis in blastocysts was not affected by dietary treatments [133]. 

Overall, the consensus favours the notion that dietary fat supplementation has limited value in alleviating the deleterious effect of negative energy balance on oocyte developmental competence [134]. Indeed, there is little information in bovine OPU-IVEP models to advocate the usefulness of lipid supplementation to improve in vitro embryo production in cattle that are metabolically challenged. As such, there are no clear data supporting the use of fatty acid supplementation as an effective way of improving the oocyte developmental competence of oocyte donors in cattle. 

## 4. Impact of Mineral and Vitamin Supplementation of Bovine Oocyte Donors on In Vitro Embryo Production

Few studies have explored the effect of micronutrients in bovine OPU-IVEP models. Hidalgo et al. [135] found that retinol injections to Asturian Valley heifers enhanced in vitro blastocyst production, but resultant embryos were unable to achieve pregnancy following embryo transfer. Interestingly, this detrimental effect was associated with the accumulation of retinol in follicular fluid that promoted blastocyst formation but somehow impaired their potential for implantation [135]. In *Bos indicus* cattle (i.e., Gyr, Brahman, and Nellore cows), in vitro blastocyst yield was improved with an injection of vitamin A and E [136]. But given the inability of embryos derived from retinol-exposed oocytes to achieve pregnancy [135], the potential beneficial effect of vitamin supplementation to truly improve oocyte developmental competence from oocyte donors remains uncertain.

Gomes da Silva [137] compared proteinated trace minerals (i.e., minerals bound to amino acids and peptides) with inorganic trace minerals formulated to meet or exceed trace mineral requirements, and found that Holstein cows provided with these treatments from 30 days before to 56 days after calving did not show differences in in vitro embryo production. Another study comparing the supplementation of complexed trace minerals with inorganic forms of trace minerals in Angus cows did not find a significant effect on the cleavage rate and progression to the blastocyst stage. However, an odds ratio analysis revealed that oocytes from cows supplemented with complexed trace minerals had an increased likelihood of producing a transferable embryo [138]. Whether this nutritional approach will work in dairy cows remains to be determined. 

The testing of vitamins and minerals to improve oocyte developmental has not been applied in oocyte donors with substantial micronutrient deficiencies, and this might partially account for the lack of a clear effect on in vitro embryo production. Overall, there is not enough unambiguous data available to promote the supplementation of vitamins and minerals as a means to increase the IVEP of bovine oocyte donors with no apparent micronutrient deficiencies.

## 5. Experimental Considerations in Nutritional Research with Bovine OPU-IVEP Models

The standard of in vitro embryo production achieved by the research team is relevant to consider in OPU-IVEP studies addressing the effect of oocyte donor nutrition on oocyte developmental competence. The IVEP outcome is determined to a great extent by the OPU technique (including operator skills and equipment) and the embryologist involved in the study. Early research examining bovine oocyte donor nutrition achieved a blastocyst production from oocytes cultured ranging from 5 to 11%, rendering it difficult to detect a clear effect on IVEP [139]. Indeed, studies reporting less than 12% blastocyst formation (from oocytes cultured) among their experimental groups did not detect effects of nutritional supplementation on IVEP [22,116,139]. Nevertheless, in some instances, low blastocyst production can be improved by nutritional interventions, as shown by the increase from 12 to 24% in blastocyst production in overfed growing heifers that were subjected to dietary restriction [38]. This does not mean that a cutting point of 12% for blastocysts production needs to be achieved in nutritional studies using OPU-IVEP models, but efforts should be implemented to ensure control animals reach an embryo production akin with the 20–30% blastocyst production achieved with the current OPU-IVEP protocols from established commercial enterprises [17,140]. Not many research institutions have the resources and staff to carry out OPU-IVEP cycles on a regular basis that will allow them to develop the necessary expertise to achieve a good standard in in vitro embryo production. Academic institutions could address this issue with collaborations with well-established commercial OPU-IVEP companies in order to provide data of practical relevance.

Significant changes in the blood concentrations of metabolic hormones and metabolites associated with metabolic stress, such as negative energy balance in dairy cows, do not always result in decreased IVEP [39,141]. This could be partially explained by the fact that analyte concentrations in ovarian follicular fluid are not always reflected in peripheral circulation, which has been proposed to be the result of unknown maternal adaptations that aim at promoting a nutritional microenvironment favourable for oocyte development [71]. As such, an important consideration during nutritional studies in OPU-IVEP models is the ability of dietary interventions to alter the nutrient composition in follicular fluid as this will ultimately impact oocyte quality. Although a technically challenging procedure, especially for small–medium follicles, the collection of follicular fluid for the determination of analyte concentrations is pivotal to better understand the effects of nutritional challenges and/or supplements on oocyte developmental competence.

It is important to report blastocyst production based on the number of oocytes cultured rather than just on inseminated or cleaved oocytes as this gives an indication of the robustness of the OPU/IVEP model. Also, the majority of OPU-IVEP studies do not report the capacity of the resultant embryos to achieve pregnancy following embryo transfer to suitable recipients. Although logistically and financially challenging, examination of the capacity of resultant embryos to achieve pregnancy to term is essential to truly determine the effect of nutritional treatments on oocyte donors upon the developmental competence of their oocytes. The relevance of conducting embryo transfer studies is illustrated by the above-mentioned enhanced in vitro embryo yield achieved by treating oocyte donors with retinol, but that impaired the capacity of the resultant embryos to achieve implantation [135].

Consideration of the initial BCS of cows and heifers and its monitoring during experimental trials has shown to be a simple but effective way to determine the overall metabolic effect of experimental feeding regimes. Indeed, the absence of significant changes in the BCS of oocyte donors sometimes seems to be a better determinant of the lack of impact of nutritional treatments on IVEP rather than blood concentration changes in metabolic hormones and metabolites [63,81]. However, BCS is a subjective assessment of body fat deposition, and it is influenced by the experience level of the evaluator [142]. As such, subtle changes in BCS might be difficult to detect just by visual observation [143]. Nevertheless, the accuracy of BCS measurements could be improved with the use of automated systems [144].

There are several technical factors that can impact the efficiency of OPU-IVEP cycles, but aspects related to animal well-being should also be considered. Indeed, animal comfort, pen size, and housing are experimental elements that should be taken into account when developing nutritional trials in cattle [145]. Given that psychological stress can impair reproductive function [146], including oocyte quality [103,104], the possible stress response associated with the OPU procedure and the taking of other samples should be considered, attempting to minimize severity (e.g., liver biopsy) and duration (serial venepuncture) of sampling along with a reduction in sampling time points.

## 6. Conclusions

The preceding discussion indicates that the harmful effects of overnutrition on in vitro oocyte developmental competence are dependent to a great extent on the duration of overfeeding and the initial BCS of oocyte donors. Overall, the available evidence suggests that oocyte donors with moderate BCS have an increased chance of providing a metabolic microenvironment in ovarian follicles that will promote blastocyst formation in vitro. The use of fatty acid and micronutrient supplementation to improve IVEP in cattle is debatable with the current information available. As such, the supply of maintenance nutritional requirements according to developmental and productive stages seems to be enough to provide oocyte donors with a good chance of producing embryos. Finally, besides technical factors, animal well-being aspects need be considered in nutritional experiments using OPU-IVEP in cattle.

## Figures and Tables

**Table 1:** Selected list of studies published in the last ten years showing worldwide variation in in vitro embryo production coupled with ovum pick-up in cattle.

Donor Type ^1^ (*n*)	No. of OPUs ^2^	OVS ^3^	Embryo Rate ^4^ (Day) ^5^	Embryos per OPU ^6^	Country	Ref.
Dairy-C (*n* = 32)	NR	N	8–14% (D8)	NR	France	[39]
Dairy-C/H (NR)	1285	N	23–34% (D7)	1.06–1.73	Netherlands	[40]
Dairy-C (*n* = 15)	20	N	8–15% (D7–8)	1.8–2.28	Israel	[41]
Dairy-C/H (NR)	2817	NR	17–19% (NR)	1.43–2.49	Italy	[42]
Dairy-C/H (*n* = 59)	NR	N	19–20% (D7)	1.2–3.0	Brazil	[43]
Dairy-C (*n* = 30)	60	Y/N ^**b**^	10–52% (D7)	1.0–4.4	Brazil	[18]
Dairy-C/H (*n* = 26)	134	Y	33–62% (D7)	2.9–7.3	USA	[32]
Dairy-H (*n* = 16)	32	Y	36–50% (D7) ^**c**^	NR	France	[44]
Dairy-H (*n* = 9)	54	N	10–16% (D7) ^**c**^	NR	Brazil	[21]
Dairy-H (*n* = 64)	202	NO	17% (D7)	1.1–1.4	Germany	[45]
Dairy-H (*n* = 90)	NR	Y/N ^**b**^	25–30% (D7)	2.4–4.7	Brazil	[46]
Dairy-H (*n* = 9)	81	Y/N ^**b**^	46–71% (D8) ^**c**^	5.6 ^**d**^	UK	[47]
Dairy-C (*n* = 35)	35	Y	21–38% (D7)	4.1–5.6	Brazil	[48]
Dairy-C (*n* = 15)	240	N	26–42% (D8)	NR	Germany	[49]
Dairy-H (*n* = 64) ^**a**^	64	Y	33–48% (D7)	NR	Brazil	[50]
Dairy-H (*n* = 41)	41	Y	0–38% (D7)	NR	Iran	[51]
Dairy-H (*n* = 20)	110	Y	36–65% (D8) ^**c**^	NR	UK	[52]
Beef-H (*n* = 34)	NR	N	27–33% (D7)	2.2–7.0	Brazil	[43]
Beef-C (*n* = 6)	32	Y	43–47% (D7)	3.3–3.8	USA	[32]
Beef-H (*n* = 9)	54	N	28% (D7)	NR	Brazil	[21]
Beef-H (*n* = 43)	NR	Y/N ^**b**^	51–62% (D8)	NR	Brazil	[53]
Beef-C (*n* = 19)	152	N	32–47% (D7)	1.5–4.3 ^**d**^	S. Korea	[54]
Beef-C (*n* = 11)	55	N	2.7–50% (D8)	0.4–6.4 ^**d**^	Japan	[55]
Beef-C (*n* = 66)	NR	N	13–41% (D7)	0.6–18.4	Brazil	[56]
Beef-C (*n* = 36)	432	N	33–34% (D7)	5.4	Brazil	[31]
Beef-C (*n* = 2)	16	Y	22–39% (D8)	NR	Japan	[57]
Beef-C (*n* = 32)	224	N	28–49% (D7)	1.7–10.3 ^**e**^	Brazil	[58]
Beef-C (*n* = 20)	31	N	7–33% (D7) ^**c**^	NR	USA	[59]
Beef-C (*n* = 18)	32	N	12–14% (D7) ^**c**^	NR	USA	[60]
Beef-C (*n* = 18)	180	N	18–29% (D7)	NR	Pakistan	[61]
Beef-C (*n* = 12)	104	N	12–16% (D7) ^**c**^	NR	Pakistan	[62]
Beef-C (*n* = 6)	18	N	36–41% (D7–8)	NR	Japan	[63]

**^1^** Cow (C) or post-pubertal heifer (H); **^2^** number of OPU sessions; **^3^** OV = gonadotrophin treatment for ovarian stimulation; **^4^** embryos produced from cultured oocytes; **^5^** Day = day embryo production was evaluated (day 0 = day of IVF); **^6^** mean number of viable embryos produced per OPU session; ***n*** = number of animals; NR = not reported; N = no; Y = yes; **^a^** = pregnant heifers were used in the study. **^b^** = The study included non-superstimulated and superstimulated animals; **^c^** = authors used inseminated rather than cultured oocytes for calculation; **^d^** = calculated when total number of embryos produced and number of OPU sessions were available in the article (no. of embryos produced/no. of OPU sessions); **^e^** = values are from one of the experiments performed in the study with 16 oocyte donors and 96 OPU sessions.

**Table 2:** Summary of studies investigating the effects of fatty acid supplementation of lactating dairy cows on in vitro embryo production.

Diet Treatments	Time of Treatment ^1^	COC Quality	Blastocyst (B) Production			Embryo Quality	Ref.
				B/IVF	B/Cleaved		
Sunflower oil Ca salts of trans FA Ca salts of VO Linseed oil (LO)	−21 to +107	↔ oocyte apoptosis	↔	8.4% 6.9% 2.0% 5.2%	13.1% 9.2% 3.0% 9.1%	↔ cell number ↔ apoptosis	[116]
Rumen inert fat—low Rumen inert fat—high	+40 to +60	↔ morphology grade	↑	19.4% 27.4%	29.1% 38.0%	↑ cell number	[117]
Rumen inert fat Soya Linseed	+46 to +125	↔ morphology grade	↔	20.0% 19.0% 19.0%	29.0% 30.0% 32.0%	↔ cell number ↔ apoptosis	[118]
Saturated fat Flaxseed oil Fish oil	−24 to +100	↑↓ FA composition	↔	8.8% 15.2% 13.4%	NR NR NR	NR	[41]
Sunflower oil Palm oil No supplement	−28 to +111	↑ morphology grade	↔	17.6% 20.7% 18.6%	NR NR NR	NR	[119]
Whole raw soybean	−90 to 0	↔ morphology grade	↔	10–17%	NR	NR	[120]
Fish oil Soya oil	+77 to +141	↑↓ oocyte lipidome	↔	NR NR	49.6% 42.3%	↑ Morphological quality	[121]
Coconut oil LO + safflower oil (SaO) Lutalin ® Lutalin ® + LO + SaO	−63 to +56	NR	↔	NR NR NR NR	23.5% 17.4% 26.1% 15.2%	↔ cell number	[122]
Prilled saturated fat Sea buckthorn oil	−20 to +67	↔ GC gene expression	↔	34% * 39% *	NR NR	↔ lipid accumulation	[123]

^1^ = days before (−, prepartum) and after (+, postpartum) calving (day 0), COC = cumulus–oocyte complex, Ca = calcium, FA = fatty acids, VO = vegetable oils, Lutalin^®^ = conjugated linoleic acid, NR = not reported, B/IVF = calculated on the number of in vitro fertilised oocytes, B/IVF = calculated on the number cleaved embryos, GC = granulosa cells. * Reported morula production not blastocyst formation rate. ↑ = increase, ↓ = decrease, ↔ = no effect, ↑↓ = increases and decreases in analytes were observed.

## Data Availability

Not applicable.
